# Alarm bells or echoes of hope? A new perspective on the global youth mental health crisis

**DOI:** 10.1017/S0033291725102249

**Published:** 2025-11-03

**Authors:** Levi van Dam, Jim van Os, Geert Jan Stams, Hans Ormel

**Affiliations:** 1 Department of Child Development and Education, University of Amsterdam, Amsterdam, the Netherlands; 2 Dutch Innovation Network for Societal Youth Challenges, Garage2020, Amsterdam, The Netherlands; 3 Division of Neuroscience, University Medical Center Utrecht, Utrecht, the Netherlands; 4 King’s Health Partners, Department of Psychosis Studies, Institute of Psychiatry, King’s College London, London, UK; 5 Department of Psychiatry, Faculty of Medical Sciences, University of Groningen, Groningen, the Netherlands

**Keywords:** Population mental health, Children and adolescents, Global burden of disease, Prevention

## Abstract

The youth mental health debate is often framed in alarming terms, yet evidence for a recent surge in mental disorder prevalence remains inconclusive. We argue that much of the apparent increase relies on self-report data, and thus may reflect heightened awareness of mental problems in youth themselves. Long-term epidemiological studies indicate relative stability or even decline until the COVID-19 pandemic, after which rates of anxiety and depression rose sharply. At the same time, indicators of youth development – including reduced school drop-out, unemployment, delinquency, and adversity – suggest more positive trajectories than the crisis narrative implies. We call for a shift beyond the disorder–distress dichotomy, recognizing the increased visibility and vocalization of emotional difficulties among adolescents as a positive sign, potentially reflecting adaptive coping rather than pathology. Such reframing will steer novel solutions that focus on promoting well-being and understanding what keeps youth healthy, rather than just treating illness.

The current debate around youth mental health is filled with alarming statements. For example, the opening paragraph of the *Lancet Psychiatry* Commission on youth mental health (McGorry et al., 2024) argues: ‘*Mental ill health, which has been the leading health and social issue impacting the lives and futures of young people for decades, has entered a dangerous phase. The youth mental health crisis is more than a warning; it might be our last chance to take action’.* Although we fully endorse the Commission’s statement that *a negligent, or worse, a denialist response would be unacceptable and harmful*, we question whether these statements are justified. As Janet Currie of the National Bureau of Economic Research states: ‘the idea that the current child mental health crisis is a recent and unprecedented phenomenon is a myth, the available sources suggest that the child mental health crisis has existed for decades and that the number of children with an underlying mental health condition has been high and relatively stable over time’ (Currie, 2025). Given these opposing positions, we draw attention to two critical issues in the current debate: 1) the data on which the statements are based and their interpretation; 2) the neglect of data on youth development, such as school drop-out, youth-unemployment, and sexual abuse, to look at the social function of youth in society, and not only at psychopathology. Taking these factors into account leads to a new perspective: the current generation of youth is more open about mental health, driving solutions that focus on promoting well-being and understanding what keeps youth healthy, rather than just treating illness.

Our first critique addresses the difference between two types of data that are used: epidemiological data using clinical diagnostic interviews to assess psychiatric disorders and self-report questionnaires to assess mental health complaints (distress). For example, the *Lancet Psychiatry* Commission on youth mental health highlights findings from the UK in which a recent study (Armitage et al., 2023) indicated that the ‘*emotional problems are emerging earlier in the lifespan and showing a trend toward increasing prevalence as indicated in a study comparing two cohorts a decade apart’.* However, this study used the parent-rated emotional subscale of the Strengths and Difficulties Questionnaire (SDQ-E). Although the SDQ-E shows moderate to high accuracy in assessing depressive and anxiety disorders (Armitage et al., 2023), Richter et al. (2019) found that the small increase in mental ill health (in adults) was only significant if measured by means of self-report questionnaires, but not if assessed with clinical diagnostic interviews. This suggests that the increase in self-reported mental health problems may be explained by increased mental health awareness instead of an increase in mental disorder prevalence (Foulkes & Andrews, 2023). Since questionnaires can be affected by differences in perception and interpretation of items, cohort comparisons based on questionnaires can only be validly conducted after measurement invariance has been established over time. However, no measurement-invariance was established when using the SDQ-E in different cohorts, making cross-time comparisons of emotional symptoms highly problematic.

The commission also highlights findings from Australia: ‘*More alarmingly, the recent national study of mental health and wellbeing in Australia (2020–22) showed a 50% increase in prevalence of diagnostic-level mental disorders in people aged 16–24 years since 2007, reaching an annual prevalence rate of 39% in 2020–22’.* However, the Commission does not mention that the data from the 2007 cohort were obtained with a 10-item questionnaire (Kessler et al., 2002), whereas the 2020–2022 data were obtained through clinical interviews. Therefore, a valid comparison cannot be made due to possible instrumentation bias (Cook et al., 1979), while caution is warranted when comparing mental health issues with mental health disorders.

If we inspect the available data on the prevalence of mental disorders, investigating changes over the past three decades, a slightly different picture emerges. For example, Piao et al. (2022) examined changes in global rates of mental illness among youth (0–20 years). They reported that mental disorder prevalence decreased slightly by 1.3% from 1990 to 2019, whereas the burden of mental disorders significantly increased by 14.9%, mostly in Western countries. Liu et al. (2025) report a stable worldwide age-standardized rate for total mental disorders among children and adolescents from 1990 to 2019. However, a notable increase was observed between 2019 and 2021, corresponding with the COVID-19 pandemic. Among the 10 types of mental disorders, anxiety disorders and depressive disorders showed sharp upward trends compared with total mental disorders. In an unpublished pre-print based on data from the Global Burden of Disease study, Zhang et al. (2025) conclude that from 1990, the prevalence of psychiatric disorders indicated an overall decreasing trend in total of 3.31% until 2019. However, after 2019, the prevalence of psychiatric disorders rapidly increased from 2019 to 2021, with 9.07%, particularly for anxiety and depressive disorders. A meta-analysis of the prevalence of mental disorders among 80,879 adolescents in 29 samples showed that during the COVID-19 pandemic, the prevalence of depression and anxiety was 25.2% and 20.5%, respectively (Racine et al., 2021). Since data after COVID-19 (e.g., 2023 and beyond) is lacking, it remains unclear whether this increase in depression and anxiety is an adequate response to a stressful situation and therefore declines after 2023, or if this unique increase, followed by a 30-year period of decline, remains. However, the Dutch NEMESIS prevalence study (Ten Have et al., 2023) revealed that students aged 18–34 years showed a stronger increase between 2007–2009 and 2019–2022 in the prevalence of any 12-month disorder than adults aged 35 and older.

It is remarkable that in several Western countries the utilization of treatment for youth has substantially increased (Mojtabai & Olfson, 2020; British Medical Association, 2025; Dutch Youth Institute, 2025) over the past decades, while there is no robust evidence that their mental health has deteriorated. The current situation of treatment and prevention of mental problems in youth resembles the treatment-prevalence paradox (Ormel et al., 2022), namely, the paradoxical situation that more treatment does not appear to result in a substantial decrease of mental health problems, despite the small to moderate effectiveness of treatment (Weisz et al., 2017). Increased supply of (new) treatments may have brought about more demand, or in terms of Say’s general economic law of markets: supply creates its own demand. The failure of current treatment and prevention to substantially reduce the mental ill health burden at the population level may be rooted in the deeper (distal) causes (sources) of mental ill health, which are rarely addressed, neither in treatment nor in prevention, including social and economic deprivation, problematic parenting, lack of education, living in disadvantaged neighborhoods, exposure to unregulated social media, insecurity of employment, and the negative consequences of climate change (Ormel & VonKorff, 2024). To reduce the burden, governments must be prepared to tackle the deeper causes head-on. This can only succeed with political will, adequate long-term funding with effective legal and social embedding, a life-course perspective, and endurance.

To prevent blinding ourselves by only looking at distress (mental health symptoms) and (psychiatric) disorders, we advocate an ecological perspective (Bronfenbrenner, 1977). In broadening the scope to understand the social function of youth in society, we need to take into account trends in behavioral (mal)adaptation data in order to better understand the impact of mental ill health in important areas of youth development. For example, in the Netherlands, we observe a decline in school drop-out since 2010 (Dutch Youth Institute, 2025), a decline in youth unemployment since 2014 (Dutch Youth Institute, 2025) and a decline in youth involved in criminal activities since 2013 (Dutch Youth Institute, 2025), which is also observed in many other countries around the globe (Office of Juvenile Justice and Delinquency Prevention, 2025). Besides positive trends in these critical youth developmental domains, we need to look at time trends in the amount of adversity youth experience. For example, Finkelhor (2020) found that in the United States, incidence rates of Adverse Childhood Experiences (ACEs) are declining. Specifically, national population data depicting rates of parental illness, sibling death, intimate partner violence, family poverty, parental divorce, physical and sexual abuse, physical bullying, and exposure to community violence all have declined since 2000. Parental substance use (alcohol and drug use by primary parent caregiver) was the only individual ACE that was found to have increased from 2000 to 2017. Brauer et al. (2024) investigated the health consequences of exposure to risk factors from 1990 to 2021. The annualized rate of change from 1990 to 2021 for intimate partner violence was −0.2% and for childhood sexual abuse −003%, indicating a slight improvement in the level of domestic adversity youth experience. Furthermore, understanding developments in attachment styles is crucial, as they play a key role in shaping resilience during adversity by providing a strong emotional foundation that helps youth manage stress, adapt to challenges, and maintain psychological well-being (Sroufe, 2005). Global research on 50 years of infant-parent attachment trends in more than 20 countries shows a decline in avoidant attachment over time compared to secure attachment, with further stable distributions at 51.6% secure, 14.7% avoidant, 10.2% resistant, and 23.5% disorganized attachment patterns (Madigan et al., 2023).

Paradoxically, we feel that the correct answer to the trend question of possible deteriorating youth mental health may be irrelevant given the urgent need to respond timely and appropriately to the multiple strong signals emerging adults send to underscore their mental health burden. Since ultimately 1 out of 2 people face mental health disorders, of which most emerge by age 15, while 3 in 4 youth experience childhood adversity (Pace et al., 2022), it is adaptive if the youth ring the bell. Interestingly, the rise in youth-reported mental health issues may suggest that a new generation is better at expressing their mental health concerns. Therefore, given the small-to-moderate effectiveness of psychological treatment (Weisz et al., 2017) and the persistent shortage of psychotherapy to address the high prevalence of mental health conditions, it may be more appropriate to interpret the strong signals of emerging adults as emotion regulation skills put to action. For example, time trends of the ‘Swiss Multicentre Adolescent Survey on Health’ examining 16.774 participants (16- to 20-year olds) indicated that young people’s willingness to talk about mental health problems between 1992/93 and 2002 improved (Dey et al., 2016). More youth became willing to discuss mental health issues with adults or friends, while fewer avoided such conversations. This pattern was observed in both non-suicidal individuals and those who reported some level of suicidality. In fact, today’s youth ringing the bell may be a sound of hope, because they may be better equipped to express their needs by effectively managing and responding to the emotional challenges of life. Such an interpretation resonates with salutogenesis (Van Os & Guloksuz, 2024), which focuses on the promotion of health, the understanding of what keeps youth well, enhancing health-promoting factors rather than just treating diseases or addressing their (distal) causes. Nevertheless, advancing this valuable and timely debate about youth mental health must acknowledge the Thomas theorem: *‘If men define situations as real, they are real in their consequences’.*

## References

[r1] Armitage, J. M., Kwong, A. S., Tseliou, F., Sellers, R., Blakey, R., Anthony, R., … Collishaw, S. (2023). Cross-cohort change in parent-reported emotional problem trajectories across childhood and adolescence in the UK. The Lancet Psychiatry, 10(6), 509–517. 37244272 10.1016/S2215-0366(23)00175-X

[r2] Asscher, J. J., Wissink, I. B., Deković, M., Prinzie, P., & Stams, G. J. J. M. (2014). Delinquent behavior, poor relationship quality with parents, and involvement with deviant peers in delinquent and nondelinquent adolescents: Different processes, informant bias, or both? International Journal of Offender Therapy and Comparative Criminology, 58(9), 1001–1019. 10.1177/0306624X13491389. 23780846

[r3] Brauer, M., Roth, G. A., Aravkin, A. Y., Zheng, P., Abate, K. H., Abate, Y. H., … Amani, R. (2024). Global burden and strength of evidence for 88 risk factors in 204 countries and 811 subnational locations, 1990–2021: A systematic analysis for the global burden of disease study 2021. The Lancet, 403(10440), 2162–2203. 10.1016/S0140-6736(24)00933-4 PMC11120204 38762324

[r4] Breuk, R. E., Clauser, C. A. C., Stams, G. J. J. M., Slot, N. W., & Dorelijers, T. A. H. (2007). The validity of questionnaire self-report of psychopathology and parent–child relationship quality in juvenile delinquents with psychiatric disorders. Journal of Adolescence, 30(5), 761–771. 17161456 10.1016/j.adolescence.2006.10.003

[r5] British Medical Association. (2025, January). *Mental health pressures in England.* National Health Services England. Retrieved from https://www.bma.org.uk/advice-and-support/nhs-delivery-and-workforce/pressures/mental-health-pressures-data-analysis

[r6] Bronfenbrenner, U. (1977). Toward an experimental ecology of human development. American Psychologist, 32(7), 513–531. 10.1037/0003-066X.32.7.513.

[r33] Cook, T. D., Campbell, D. T., & Day, A. (1979). Quasi-experimentation: Design & analysis issues for field settings (Vol. 351). Boston: Houghton Mifflin

[r7] Currie, J. (2025). Investing in children to address the child mental health crisis (Vol. w33632). National Bureau of Economic Research.

[r8] Dey, M., Reavley, N. J., & Jorm, A. F. (2016). Young people’s difficulty in talking to others about mental health problems: An analysis of time trends in Switzerland. Psychiatry Research, 237, 159–165. 26826898 10.1016/j.psychres.2016.01.048

[r9] Dutch Youth Institute. (2025, January). *Data about youthcare.* Dutch Youth Institute. https://www.nji.nl/cijfers/jeugdzorg

[r10] Dutch Youth Institute. (2025, January). Data about school drop-out. Dutch Youth Institute. https://www.nji.nl/cijfers/voortijdig-schoolverlaten

[r11] Dutch Youth Institute. (2025, January). *Data about youth unemployment.* Dutch Youth Institute. https://www.nji.nl/cijfers/jeugdwerkloosheid

[r12] Dutch Youth Institute. (2025, January). *Data about youth delinquency.* Dutch Youth Institute. https://www.nji.nl/cijfers/delinquentie

[r13] Finkelhor, D. (2020). Trends in adverse childhood experiences (ACEs) in the United States. Child Abuse & Neglect, 108, 104641. 10.1016/j.chiabu.2020.104641. 32739600

[r14] First, M. B. (2017). Factors in the development of psychiatric epidemics. In K. S. Kendler & J. Parnas (Eds.), Philosophical issues in psychiatry IV: Classification of psychiatric illness (pp. 130–142). Oxford: Oxford University Press.

[r15] Foulkes, L., & Andrews, J. L. (2023). Are mental health awareness efforts contributing to the rise in reported mental health problems? A call to test the prevalence inflation hypothesis. New Ideas in Psychology, 69, 101010.

[r32] Kessler, R. C., Andrews, G., Colpe, L. J., Hiripi, E., Mroczek, D. K., Normand, S. L., … & Zaslavsky, A. M. (2002). Short screening scales to monitor population prevalences and trends in non-specific psychological distress. Psychological medicine, 32(6), 959–976. 12214795 10.1017/s0033291702006074

[r16] Liu, Y., Ren, Y., Liu, C., Chen, X., Li, D., Peng, J., … Ma, Q. (2025). Global burden of mental disorders in children and adolescents before and during the COVID-19 pandemic: Evidence from the global burden of disease study 2021. Psychological Medicine, 55, e90. 10.1017/S0033291725000649. 40098477 PMC12080646

[r17] Madigan, S., Fearon, R. M., van IJzendoorn, M. H., Duschinsky, R., Schuengel, C., Bakermans-Kranenburg, M. J., … Verhage, M. L. (2023). The first 20,000 strange situation procedures: A meta-analytic review. Psychological Bulletin, 149(1–2), 99–122.

[r31] McGorry, P. D., Mei, C., Dalal, N., Alvarez-Jimenez, M., Blakemore, S. J., Browne, V., … & Killackey, E. (2024). The Lancet Psychiatry Commission on youth mental health. The Lancet Psychiatry, 11(9), 731–774. 39147461 10.1016/S2215-0366(24)00163-9

[r18] Mojtabai, R., & Olfson, M. (2020). National trends in mental health care for US adolescents. JAMA Psychiatry, 77(7), 703–714. 32211824 10.1001/jamapsychiatry.2020.0279 PMC7097842

[r19] Office of Juvenile Justice and Delinquency Prevention. (2025, January). *Youth violence.* Retrieved from https://ojjdp.ojp.gov/newsletter/ojjdp-news-glance-septemberoctober-2022/data-show-decline-arrests-youth-violent-crimes

[r20] Ormel, J., & VonKorff, M. (2024). What should a nation do to prevent common mental disorders? Meet seven conditions for effective prevention. Mental Health & Prevention, 26, 200320. 10.1016/j.mhp.2024.200320.

[r21] Ormel, J., Hollon, S. D., Kessler, R. C., Cuijpers, P., & Monroe, S. M. (2022). More treatment but no less depression: The treatment-prevalence paradox. Clinical Psychology Review, 91, 102111. 34959153 10.1016/j.cpr.2021.102111

[r22] Pace, C. S., Muzi, S., Rogier, G., Meinero, L. L., & Marcenaro, S. (2022). The adverse childhood experiences–international questionnaire (ACE-IQ) in community samples around the world: A systematic review (Part I). Child Abuse & Neglect, 129, 105640. 35662684 10.1016/j.chiabu.2022.105640

[r23] Piao, J., Huang, Y., Han, C., Li, Y., Xu, Y., Liu, Y., & He, X. (2022). Alarming changes in the global burden of mental disorders in children and adolescents from 1990 to 2019: A systematic analysis for the global burden of disease study. European Child & Adolescent Psychiatry, 31(12), 1827–1845. 35831670 10.1007/s00787-022-02040-4

[r24] Racine, N., McArthur, B. A., Cooke, J. E., Eirich, R., Zhu, J., & Madigan, S. (2021). Global prevalence of depressive and anxiety symptoms in children and adolescents during COVID-19: A meta-analysis. JAMA Pediatrics, 175(11), 1142–1150. 10.1001/jamapediatrics.2021.2482. 34369987 PMC8353576

[r25] Richter, D., Wall, A., Bruen, A., & Whittington, R. (2019). Is the global prevalence rate of adult mental illness increasing? Systematic review and meta-analysis. Acta Psychiatrica Scandinavica, 140(5), 393–407. 31393996 10.1111/acps.13083

[r26] Sroufe, L. A. (2005). Attachment and development: A prospective, longitudinal study from birth to adulthood. Attachment & Human Development, 7(4), 349–367. 10.1080/14616730500365928. 16332580

[r27] Ten Have, M., Tuithof, M., van Dorsselaer, S., Schouten, F., Luik, A. I., & de Graaf, R. (2023). Prevalence and trends of common mental disorders from 2007–2009 to 2019–2022: Results from the Netherlands mental health survey and incidence studies (NEMESIS), including comparison of prevalence rates before vs. during the COVID-19 pandemic. World Psychiatry, 22(2), 275–285. 37159351 10.1002/wps.21087 PMC10168151

[r28] Van Os, J., & Guloksuz, S. (2024). Population salutogenesis – The future of psychiatry? JAMA Psychiatry, 81(2), 115–116. 38117509 10.1001/jamapsychiatry.2023.4582

[r29] Weisz, J. R., Kuppens, S., Ng, M. Y., Eckshtain, D., Ugueto, A. M., Vaughn-Coaxum, R., Jensen-Doss, A., Hawley, K. M., Marchette, L. K., Chu, B. C., Weersing, V. R., & Fordwood, S. R. (2017). What five decades of research tells us about the effects of youth psychological therapy: A multilevel meta-analysis and implications for science and practice. American Psychologist, 72(2), 79–117. 28221063 10.1037/a0040360

[r30] Zhang, Z., Li, C., Qiu, T., Su, Y., Feng, Y., Chu, Y., … & Zhang, H. (2025). Global Prevalence and Disability from Mental and Substance Use Disorders Across Childhood and Adolescence, 1990-2021: A Systematic Analysis for the Global Burden of Disease Study 2021. Available at SSRN 5073196.

