# Alarm bells or echoes of hope? A new perspective on the global youth mental health crisis – CORRIGENDUM

**DOI:** 10.1017/S0033291726103742

**Published:** 2026-03-24

**Authors:** Levi van Dam, Jim van Os, Geert Jan Stams, Hans Ormel

**Affiliations:** 1 Department of Child Development and Education, University of Amsterdam, Amsterdam, the Netherlands; 2 Dutch Innovation Network for Societal Youth Challenges, Garage2020, Amsterdam, The Netherlands; 3 Division of Neuroscience, University Medical Center Utrecht, Utrecht, the Netherlands; 4 King’s Health Partners, Department of Psychosis Studies, Institute of Psychiatry, King’s College London, London, UK; 5 Department of Psychiatry, Faculty of Medical Sciences, University of Groningen, Groningen, the Netherlands

When this article was originally published in Psychological Medicine it contained an inaccuracy in the description of the Australian cohort data cited from McGorry et al. In this Editorial, the authors state that the 2007 cohort was assessed using questionnaires, whereas the 2020–2022 cohort was assessed using interviews. Upon re-examining the original sources, the authors confirm that both cohorts were in fact assessed using clinical interviews.

Given this, the section of this Editorial regarding this should read as follows:

The commission also highlights findings from Australia: ‘More alarmingly, the recent national study of mental health and well-being in Australia (2020–22) showed a 50% increase in prevalence of diagnostic-level mental disorders in people aged 16–24 years since 2007, reaching an annual prevalence rate of 39% in 2020–22’. The data from the 2007 cohort and the 2020–2022 data were obtained through clinical interviews. Therefore, a valid comparison can be made, preventing possible instrumentation bias (Cook et al., 1979); however, caution is warranted since the most recent study was conducted during the COVID-19 pandemic.

The authors apologise for this error.
